# hMAGEA2 promotes progression of breast cancer by regulating Akt and Erk1/2 pathways

**DOI:** 10.18632/oncotarget.16184

**Published:** 2017-03-14

**Authors:** Song Park, Yonghun Sung, Jain Jeong, Minjee Choi, Jinhee Lee, Wookbong Kwon, Soyoung Jang, Si Jun Park, Hyeng-Soo Kim, Mee-Hyun Lee, Dong Joon Kim, Kangdong Liu, Sung-Hyun Kim, Zigang Dong, Zae Young Ryoo, Myoung Ok Kim

**Affiliations:** ^1^ School of Life Science, BK21 Plus KNU Creative Bio Research Group, College of Natural Sciences, Kyungpook National University, Buk-ku, Daegu 41566, Republic of Korea; ^2^ Institute of Life Science and Biotechnology, Kyungpook National University, Buk-ku, Daegu 41566, Republic of Korea; ^3^ China-US(Henan) Hormel Cancer Institute, Zhengzhou, Henan 450008, China; ^4^ The School of Animal BT Science, Kyungpook National University, Sangju-si, Gyeongsangbuk-do 37224, Republic of Korea

**Keywords:** hMAGEA2, breast cancer, triple-negative breast cancer, metastasis, Akt

## Abstract

Breast cancer is the most abundant cancer worldwide and a severe problem for women. Notably, breast cancer has a high mortality rate, mainly because of tumor progression and metastasis. Triple-negative breast cancer (TNBC) is highly progressive and lacks the expression of estrogen receptor (ER), progesterone receptor (PR), and human epidermal growth factor receptor 2 (HER2). Therefore, there are no established therapeutic targets against TNBC. In this study, we investigated whether the expression of human melanoma-associated antigen A2 (MAGEA2) is associated with TNBC. We found that hMAGEA2 is significantly overexpressed in human TNBC tissues; we also observed oncogenic properties using TNBC cell lines (MDA-MB-231 and MDA-MB-468). The overexpression of hMAGEA2 in MDA-MB-231 cell line showed dramatically increased cellular proliferation, colony formation, invasion, and xenograft tumor formation and growth. Conversely, knockdown of hMAEGA2 in MDA-MB-468 cell line suppressed cellular proliferation, colony formation, and xenograft tumor formation. Additionally, we showed that hMAGEA2 regulated the activation of Akt and Erk1/2 signaling pathways. These data indicate that hMAGEA2 is important for progression of TNBC and may serve as a novel molecular therapeutic target.

## INTRODUCTION

Triple-negative breast cancer (TNBC), which accounts for approximately 15% of breast cancer in women, is defined by lack of estrogen receptor (ER), progesterone receptor (PR), and human epidermal growth factor receptor 2 (HER2) [1–4]. According to clinical and pathological features, TNBC is the more aggressive subtype, having a more metastatic phenotype and higher rate of relapse compared with those of other breast cancers. Because of the absence of receptor expression, only traditional chemotherapeutic agents are available for the treatment of TNBC [1, 4]. Thus, it is important to identify new targets that can be used for selective therapy against TNBC.

Breast cancer is strongly influenced by dysregulation of the Akt and mitogen-activated protein kinase (MAPK) signaling cascades. Phosphorylation leads to aberrant signaling that accelerates cell proliferation as well as metastasis and cellular survival [5–13]. Phosphorylation of Akt kinase is significantly higher in TNBC [14]. Additionally, extracellular signaling-related kinase (ERK), a member of the MAPK pathway, is expressed at higher levels in TNBC [15]; ERK plays a critical role in cell proliferation and differentiation, promotes epithelial-mesenchymal transition (EMT), and accelerates cell migration by affecting contact between cells and the extracellular matrix (ETM) [6, 8, 16]. Lymph node metastasis is associated with high levels of ERK [17], while MAPK activation in TNBC is linked with a higher rate of recurrence [18], indicating that the Akt and ERK signaling pathways play an important role in progression of TNBC.

The human melanoma-associated antigen A2 (*hMAGEA2*) genes are a sub-family of cancer/testis antigens (CTAs). The *hMAGEA* family of genes is composed of *hMAGEA1* to *hMAGEA12* [19, 20]. Because of their high sequence homology, MAGEA proteins are considered functionally redundant [21]. hMAGEA proteins are expressed in the trophectoderm, developmental stage, and various cancer cells but not in normal somatic cells [19–21]. For this reason, hMAGEA families are thought to critically affect various cancers and are being investigated as potential prognostic markers in cancer patients [22–31]. In a recent study on molecular mechanisms, hMAGEA2 was shown to play an anti-apoptotic role and promote malignancy by interfering with the function of PML/p53 [32]. hMAGEA2 is also upregulated in tamoxifen-resistant breast cancer and plays a role in its development [33]. Furthermore, hMAGEA2 directly interacts with p53 and attracts histone deacetylase 3 (HDAC3) [34–37]. These findings indicate that hMAGEA2 may influence the progression of TNBC.

This study used the human TNBC cell lines (MDA-MB-231 and MDA-MB-468) to explore the cellular and molecular mechanisms underlying the invasiveness and progression of TNBC. We additionally used the TNBC human tissue array to examine the correlation between progression of TNBC and expression of hMAGEA2. hMAGEA2 was found to accelerate the growth and invasive potential of TNBC cells by activating the Akt and Erk1/2 signaling pathways. These results suggest that hMAGEA2 contributes to the progression of TNBC and may be a novel therapeutic target for the treatment of TNBC.

## RESULTS

### Expression of hMAGEA2 in human TNBC tissues

**Figure 1:**
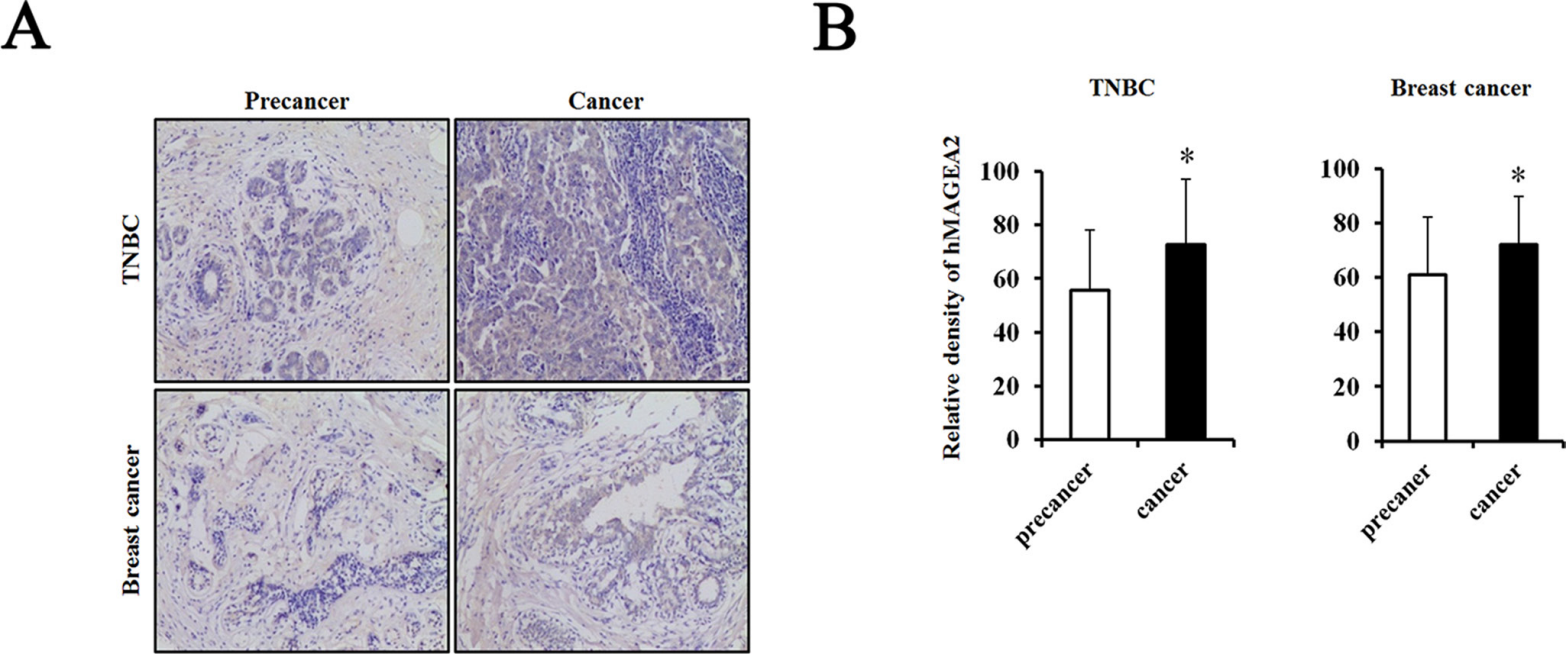
hMAGEA2 is overexpressed in human triple-negative breast cancer (TNBC) tissues and breast cancer tissues with TNBC (**A**) Immunostaining of hMAGEA2 in human TNBC tissues (upper panel) and breast cancer tissues with TNBC (lower panel). (**B**) Relative density of expression of hMAGEA2 in human TNBC tissues and breast cancer tissues with TNBC. hMAGEA2 expression was higher in human TNBC tissues and breast cancer with TNBC than that in precancer tissue. (Means ± SD, *p < 0.05, compared with control).

hMAGEA2 is used as a biomarker in various cancers and is correlated with a worse prognosis [25–31]. Therefore, we confirmed the expression level of hMAGEA2 in human TNBC and breast cancer tissues using a tissue microarray. Our results showed that the expression of hMAGEA2 was significantly upregulated in human TNBC and breast cancer tissues compared with human precancer tissues (Figure 1A, 1B).

### Establishment of hMAGEA2-overexpressed TNBC cell lines

**Figure 2:**
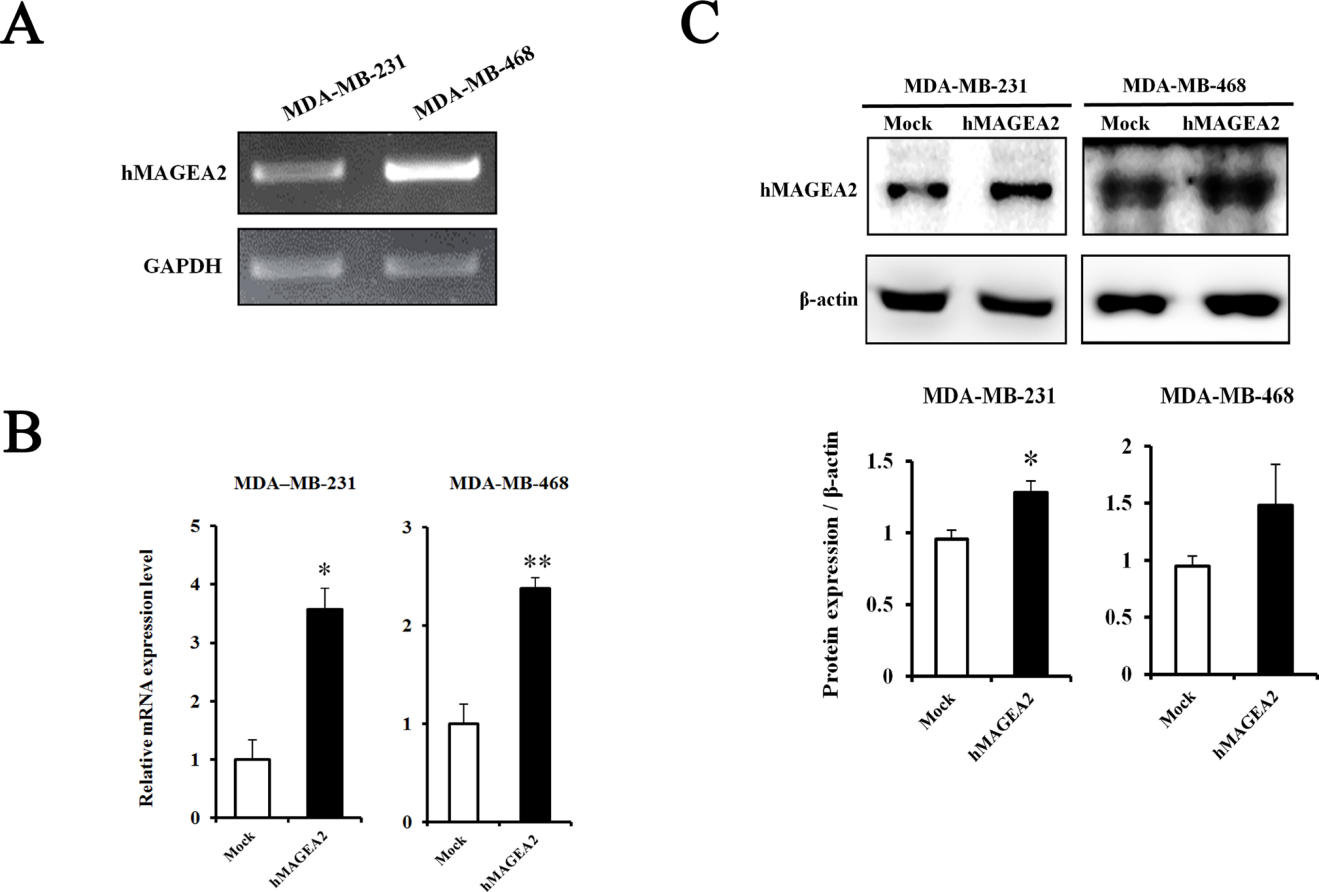
Establishment of stable overexpression of hMAGEA2 in TNBC cell lines (**A**) The level of endogenous expression of hMAGEA2 in MDA-MB-231 and MDA-MB-468 cell lines assessed using RT-PCR. (**B**) Relative mRNA expression of stably overexpressed hMAGEA2 in MDA-MB-231 and MDA-MB-468 cell lines; β-actin was used as normalization control. (**C**) Protein expression of stable hMAGEA2 overexpression in MDA-MB-231 and MDA-MB-468 cell lines; β-actin was used as loading control. (Means ± SD, *p < 0.05, **p < 0.01, compared with control).

hMAGEA2 drives tumorigenesis and progression of various cancers [21, 22, 30, 32, 34–36]. To investigate the functional relevance of *hMAGEA2* in TNBC, we established stable overexpression of hMAGEA2 in MDA-MB-231 and MDA-MB-468 TNBC cell lines. The level of endogenous expression of hMAGEA2 was confirmed in MDA-MB-231 and MDA-MB-468 cell lines using RT-PCR (Figure 2A). MDA-MB-231 cell line showed a low expression of hMAGEA2, while the expression of hMAGE-A2 in MDA-MB-468 cell line was high. Next, a plasmid vector not expressing hMAGEA2 (mock vector), and one expressing hMAGEA2, were transfected into MDA-MB-231 and MDA-MB-468 cell lines. To confirm overexpression of hMAGEA2, the levels of mRNA were measured using quantitative RT-PCR (Figure 2B). The levels of hMAGEA2 expression in MDA-MB-231 and MDA-MB-468 cell lines increased approximately 4-fold and 2.5-fold, respectively, compared with those in mock-transfected cell lines. The expression of hMAGEA2 protein was increased significantly in MDA-MB-231 cell line but MDA-MB-468 cell lines were not. (Figure 2C). All the procedures that follow were conducted using these stably transfected cell lines.

### hMAGEA2 enhanced the proliferation, colony formation, and invasion ability in TNBC cell lines

**Figure 3:**
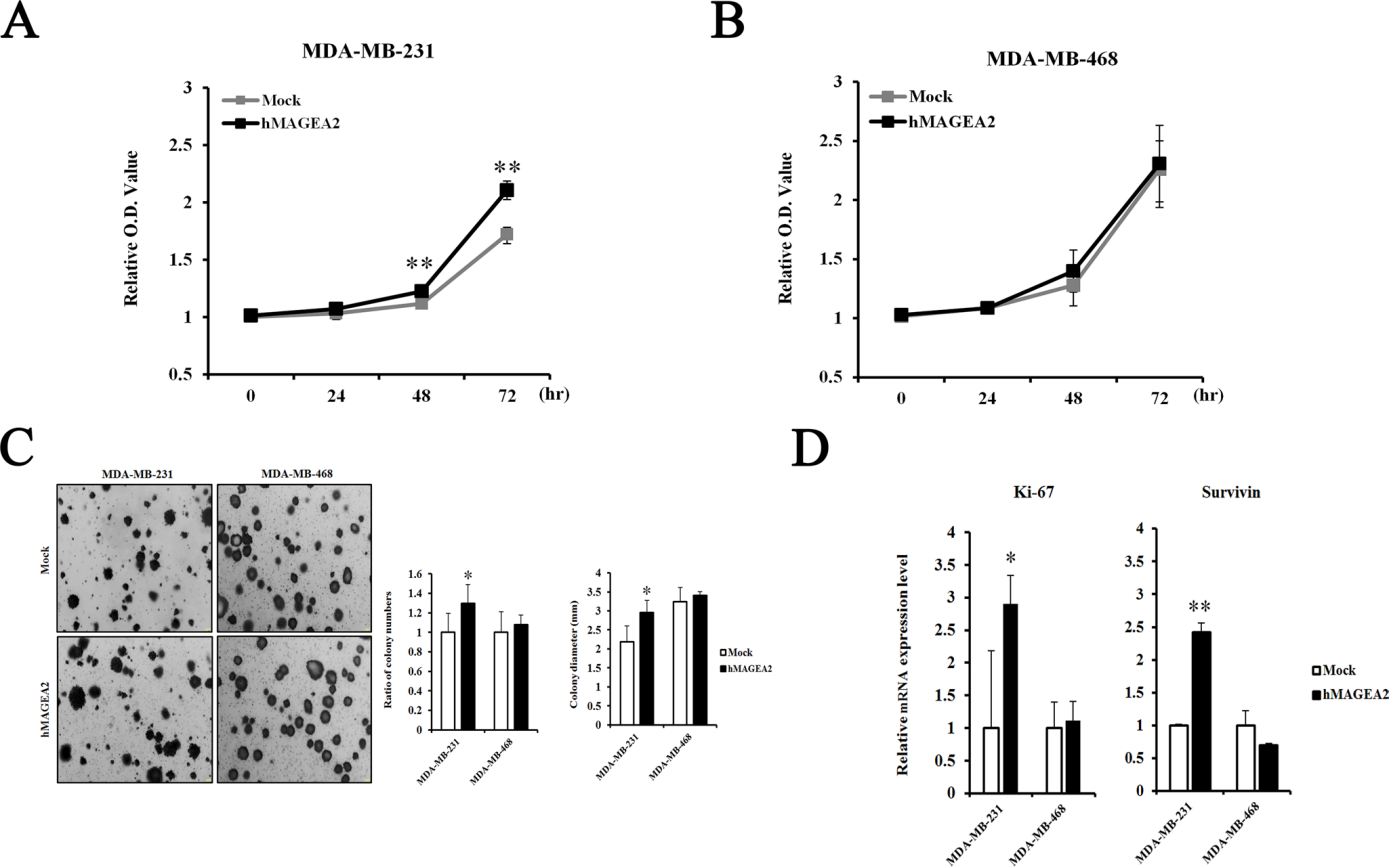
hMAGEA2 enhances proliferation capacity and anchorage-independent colony formation in TNBC cell lines (**A**) Relative optical density (O.D.) values of MDA-MB-231 cell line overexpressing hMAGEA2 were compared with those not overexpressing hMAGEA2 using the CCK-8 assay. O.D. was measured after 0, 24, 48, and 72 h. (**B**) Relative O.D. values of MDA-MB-468 cell line overexpressing hMAGEA2 were compared with those not overexpressing hMAGEA2. O.D. values were measured after 0, 24, 48, and 72 h. (**C**) Representative images of colonies were acquired using microscopy (x50 magnification, scale bar = 250 μm) (left panel) Colony numbers were counted in MDA-MB-231 and MDA-MB-468 cells lines overexpressing hMAGEA2 and those not overexpressing hMAGEA2. Colony diameters were compared in MDA-MB-231 and MDA-MB-468 cell lines stably overexpressing hMAGEA2 and those not overexpressing hMAGEA2. (right panel) (Means ± SD, *p < 0.05, compared with control) (**D**) Relative expression of Ki-67 and survivin mRNA was compared between MDA-MB-231 and MDA-MB-468 cell lines overexpressing hMAGEA2 and those not overexpressing hMAGEA2. (Means ± SD, *p < 0.05, **p < 0.01, compared with control).

**Figure 4:**
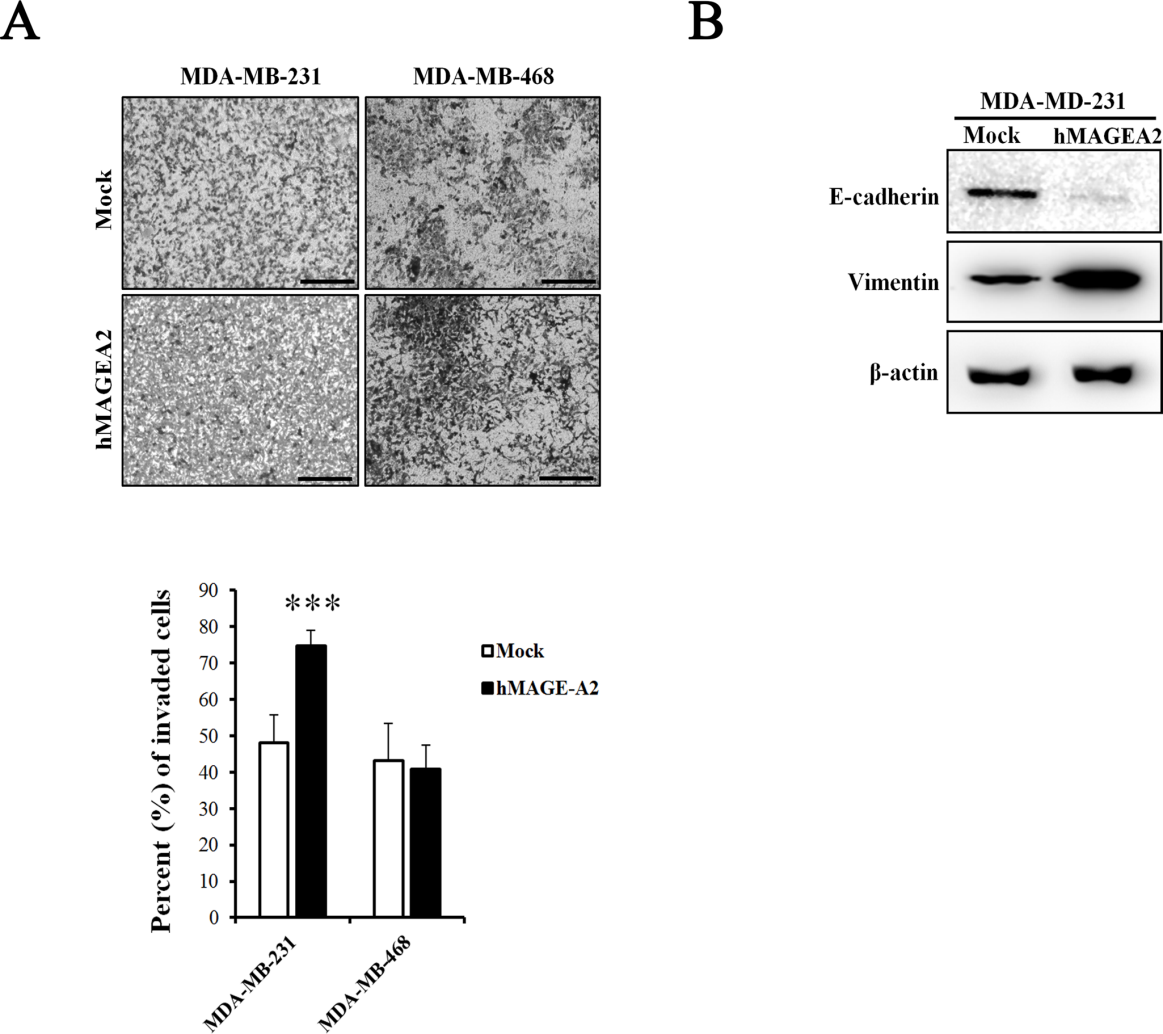
hMAGEA2 enhances metastasis ability in TNBC cell line (**A**) Representative microscopy images of invaded MDA-MB-231 and MDA-MB-468 cell lines overexpressing hMAGEA2 compared with those not overexpressing hMAGEA2 (x100 magnification, scale bar = 250 μm) (upper panel). Percentage of invaded cells (low panel) (Means ± SD, ***p < 0.001, compared with control). (**B**) Protein expression of epithelial-mesenchymal transition (EMT) markers E-cadherin and vimentin. β-actin was used as loading control.

The CCK-8 proliferation assay was used to examine the effects of the expression of *hMAGEA2*. Proliferation was measured at 0, 24, 48, and 72 h in stably overexpressed hMAGEA2 expression in MDA-MB-231 and MDA-MB-468 cells. hMAGEA2 overexpressed MDA-MB-231 cell line showed significantly increased proliferation at 48 and 72 h (Figure 3A); however, no change was observed in the proliferation of hMAGEA2 overexpressed MDA-MB-468 cell line (Figure 3B). We also assessed the levels of mRNA expression of the proliferation marker Ki-67 and anti-apoptosis marker survivin. hMAGEA2 overexpressed MDA-MB-231 cell line showed significantly increased expression of Ki-67 and survivin mRNA; however, the expression of Ki-67 and survivin mRNA in hMAGEA2 overexpressed MDA-MB-468 cell line remained unchanged (Figure 3D). Next, colony formation assay was used to investigate the effect of hMAGEA2 on anchorage-independent growth ability. In MDA-MB-231 cell line, colony number and diameter were significantly increased by overexpression of hMAGEA2 (Figure 3C). Matrigel invasion assay was used to confirm the effect of hMAGEA2 on cellular metastatic capacity. The number of invaded cells was significantly increased in the hMAGEA2 overexpressed MDA-MB-231 cell line but not in the hMAGEA2 overexpressed MDA-MB-468 cell line (Figure 4A). Additionally, the expression of epithelial marker E-cadherin was downregulated and that of mesenchymal marker, vimentin, was upregulated in the hMAGEA2 overexpressed MDA-MB-231 cell line (Figure 4B). These data suggest that hMAGEA2 accelerates metastasis via mechanisms involved in epithelial-mesenchymal transition of the MDA-MB-231 cell line. Collectively, these findings indicate that hMAGEA2 plays a key role in the progression of TNBC.

### hMAGEA2 activates the Akt and Erk1/2 signaling pathways and accelerates tumor formation

**Figure 5:**
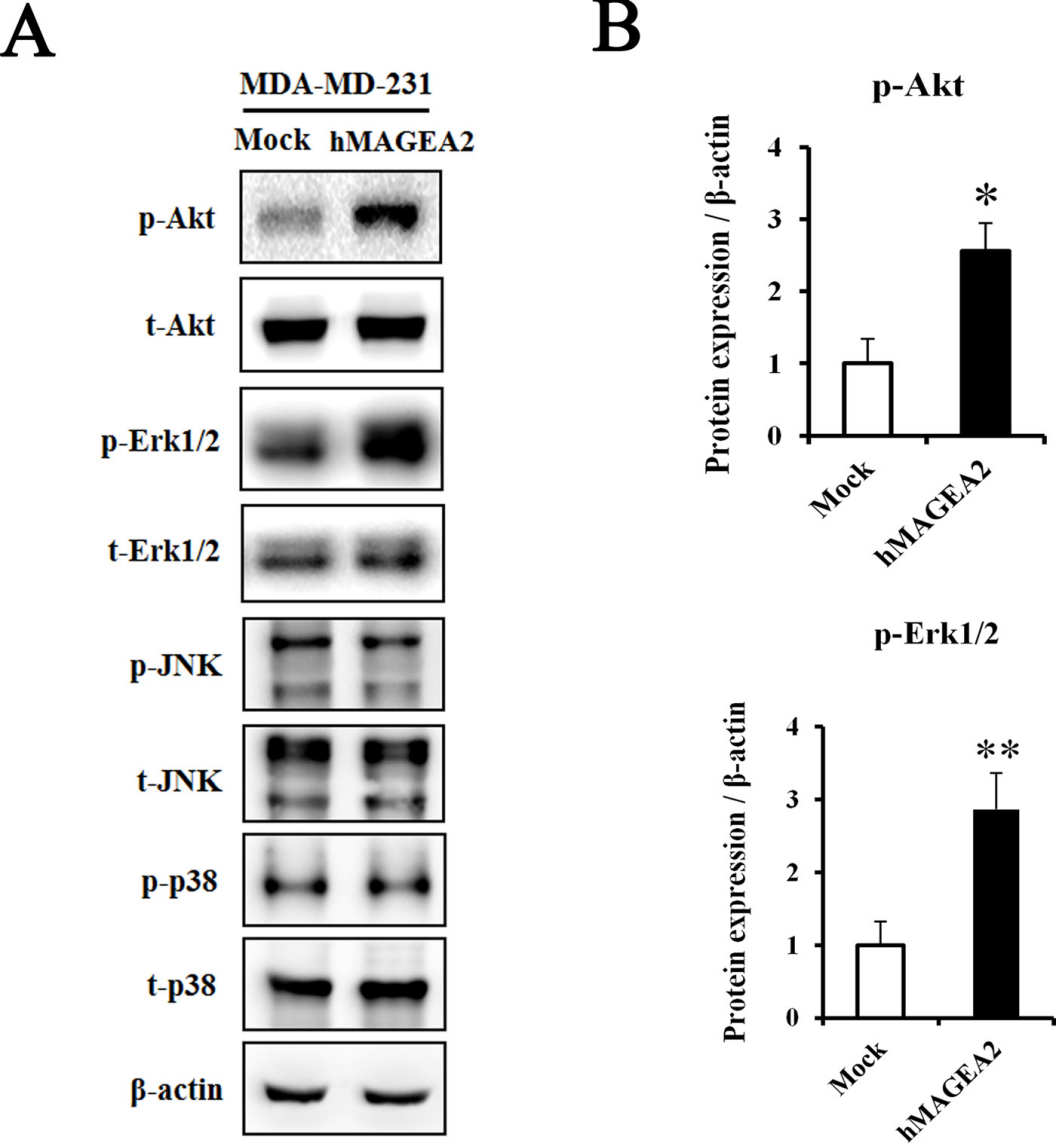
hMAGEA2 activates p-Akt and p-Erk1/2 in TNBC cell line (**A**) Protein expression of phospho-Akt, total-Akt, phospho-Erk1/2, total-Erk1/2, phospho-JNK, total-JNK, phospho-p38, and total-p38, measured in MDA-MB-231 cell line overexpressing hMAGEA2 and those not overexpressing hMAGEA2. β-actin was used as loading control. (**B**) Relative protein expression level of p-Akt and p-Erk1/2 in MDA-MB-231 cell line overexpressing hMAGEA2, and those not overexpressing hMAGEA2. (Means ± SD, *p < 0.05, **p < 0.01, compared with control).

**Figure 6:**
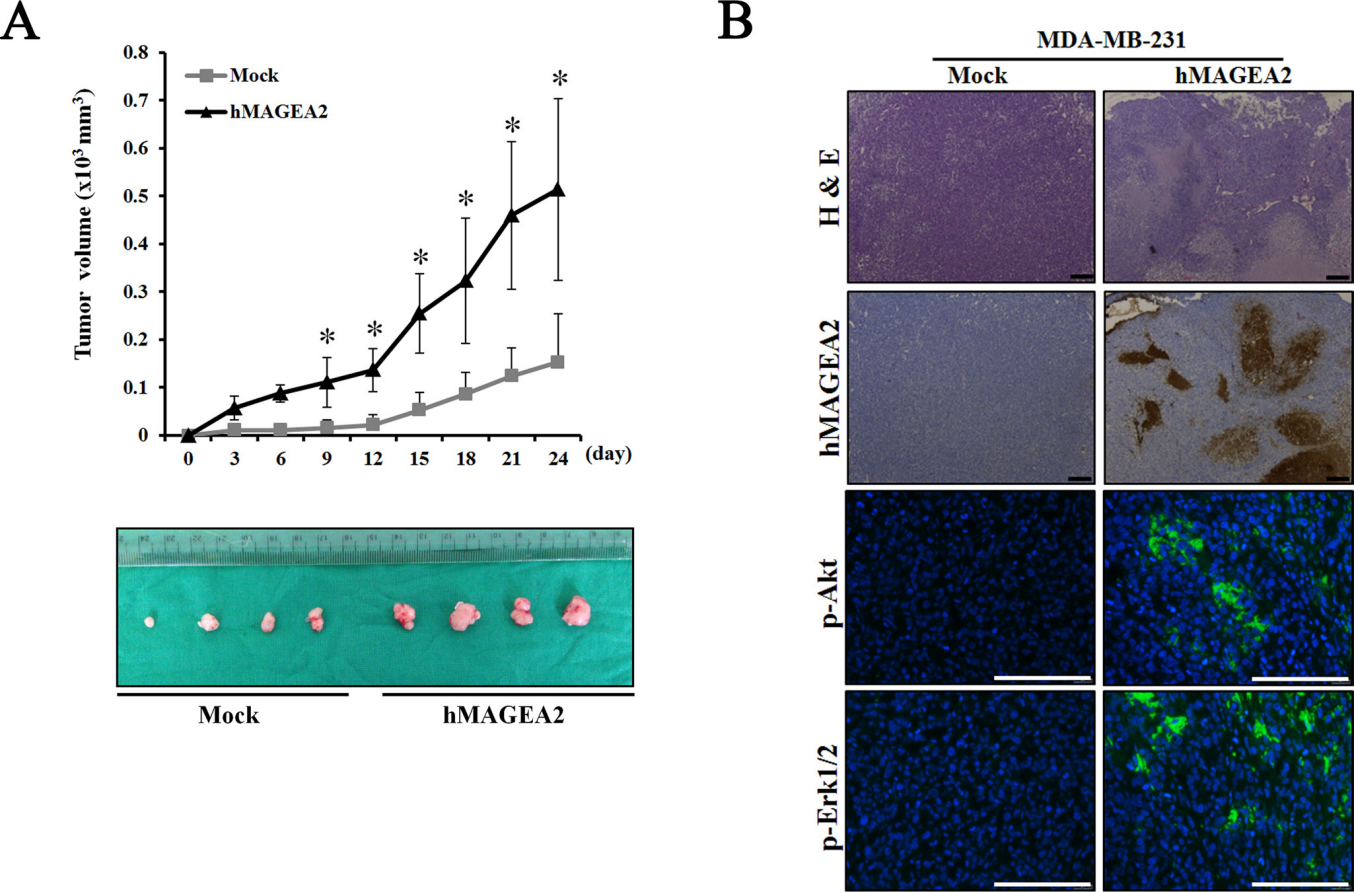
hMAGEA2 enhances xenograft tumor formation via activation of p-Akt and p-Erk1/2 (**A**) Growth curve of xenograft tumor formation after the injection of MDA-MB-231 cell line overexpressing hMAGEA2, and those not overexpressing hMAGEA2, in Balb/c nude mice. (Means ± SD, *p < 0.05) (**B**) The amount of hMAGEA2 was confirmed using hematoxylin and eosin (H&E) staining and immunohistochemistry in sectioned xenograft tumor tissues generated using MDA-MB-231 cell line stably overexpressing hMAGEA2 (x50 magnification, scale bar = 250 μm) (upper panel). The expression of phospho-Akt and phospho-Erk1/2 in xenograft tumor tissues was detected using immunofluorescence (x200 magnification, scale bar = 250 μm) (lower panel).

To assess the signaling pathway of hMAGEA2, we performed Western blotting using hMAGEA2 overexpressed MDA-MB-231 cell line. In previous studies, Akt and Erk1/2 were shown to be critical for the progression of TNBC [10, 14, 16, 18]; we sought to confirm this by examining the expression of these proteins. The expression of phospho-Akt was significantly upregulated and that of phospho-Erk1/2 was also increased in hMAGEA2 overexpressed MDA-MB-231 cell line; however, the expression of JNK and p38 in these cells remained unchanged (Figure 5A, 5B). Next, to investigate the growth rate *in vivo*, we performed a xenograft tumor formation assay. The rate of growth in tumor derived from MDA-MB-231 cell line overexpressed with hMAGEA2 was substantially increased compared with that of cells not overexpressing hMAGEA2 (Figure 6A). Xenograft tumor tissues were biopsied to compare histological morphology. Although the morphologies did not differ when examined using H&E staining, analysis using immunohistochemistry confirmed strong overexpression of hMAGEA2 in xenograft tumor tissues (Figure 6B). Furthermore, analysis using immunofluorescence confirmed the expression of phospho-Akt and phospho-Erk1/2 in xenograft tumor tissues. These data show that overexpression of hMAGEA2 in xenograft tissues significantly activated phospho-Akt and phospho-Erk1/2 (Figure 6B). These findings also indicate that overexpression of hMAGEA2 activates phospho-Akt and phospho-Erk1/2 in MDA-MB-231 cell line and tissues.

### Knockdown of hMAGEA2 repressed the progression of TNBC

**Figure 7:**
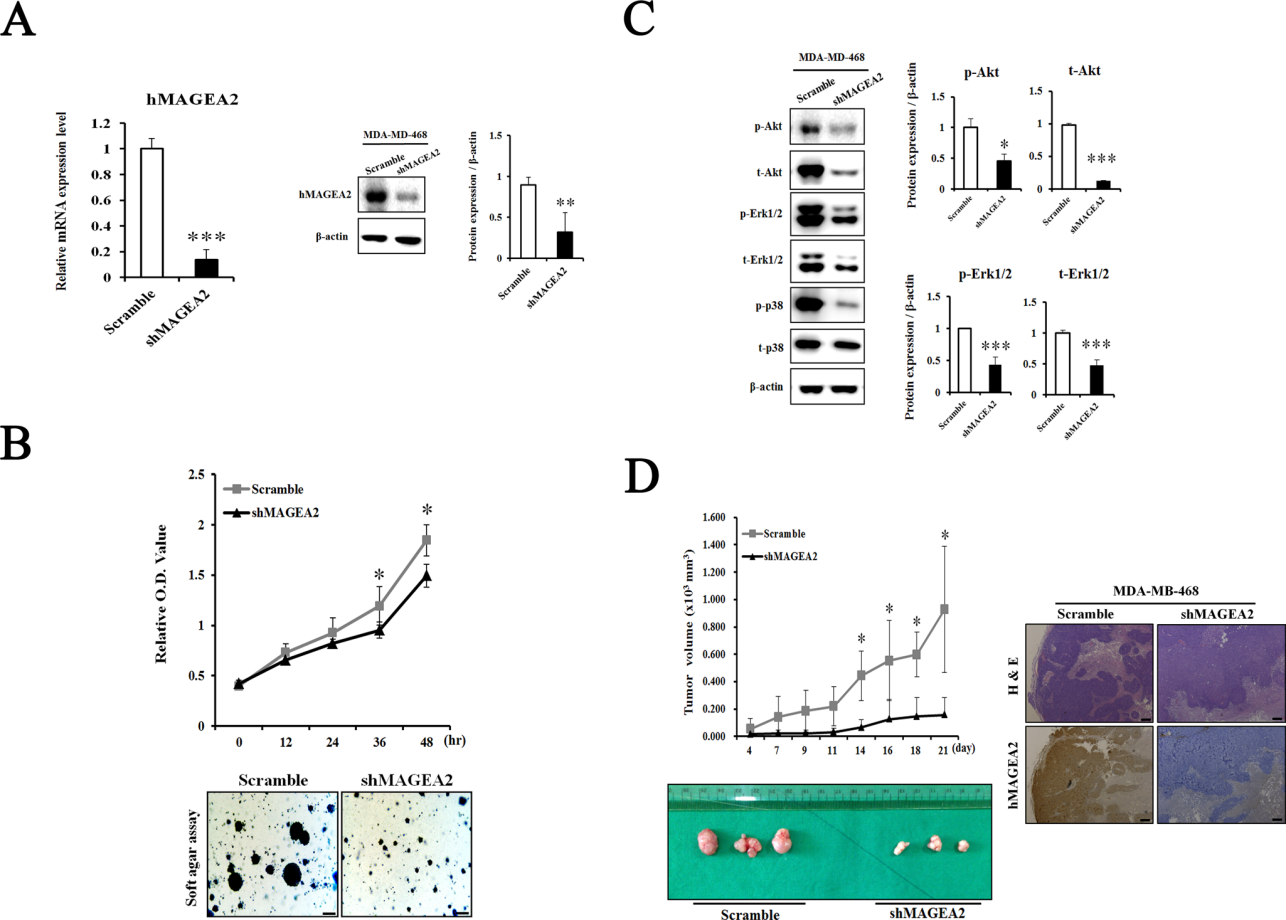
Knockdown of hMAGEA2 inhibits progression of TNBC (**A**) Establishment of hMAGEA2 knockdown in MDA-MB-468 cell line. Decrease in the expression of hMAGEA2 mRNA (left panel) and protein (right panel) compared with those of scrambled control shRNA and shMAGEA2. (**B**) Relative OD values were measured at 0, 12, 24, 36, and 48 h in scrambled control shRNA and shMAGEA2 using the CCK-8 assay (upper panel). Colony formation was compared between scrambled control shRNA and shMAGEA2 (x50 magnification, scale bar = 250 μm) (lower panel). (**C**) Protein expression of phospho-Akt, total-Akt, phospho-Erk1/2, total-Erk1/2, phospho-p38, and total-p38 was assessed in scrambled control shRNA and shMAGEA2. (**D**) Growth curve of xenograft tumor formation after injection of cell lines expressing shMAGEA2 and scrambled control shRNA in Balb/c nude mice (left panel). Using sectioned xenograft tumor tissues, H&E staining and immunohistochemistry were performed. (x50 magnification, scale bar = 250 μm) (right panel). (Means ± SD, *p < 0.05, **p < 0.01, ***p < 0.001 compared with control).

In this study, overexpression of hMAGEA2 did not alter the phenotype of MDA-MB-468 cell line. It is possible that the effects of overexpression were negated by strong endogenous expression of hMAGEA2 in these cell line. Therefore, we performed a knockdown of hMAGEA2 in the MDA-MB-468 cell line. First, we confirmed that stable knockdown of hMAGEA2 was achieved in MDA-MB-468 cell line using lentivirus infection. The expression of hMAGEA2 mRNA and protein was significantly downregulated in MDA-MB-468 cell line (Figure 7A). The results of CCK-8 proliferation assay indicated that knockdown of hMAGEA2 strongly decreased the proliferation capacity and anchorage-independent colony formation in MDA-MB-468 cell line (Figure 7B). Next, Western blot was performed to assess the expression of phosphorylated Akt and Erk1/2. Knockdown of hMAGEA2 significantly decreased the levels of phospho-Akt and phospho-Erk1/2, as well as those of total-Akt and Erk1/2. Additionally, knockdown of hMAGEA2 decreased the expression of phospho-p38 in MDA-MB-468 cell line (Figure 7C). Analysis using xenograft tumor formation assay indicated that the knockdown of hMAGEA2 resulted in slower growth rate compared with that in controls; downregulation of hMAGEA2 in xenograft tumor tissues was confirmed using immunohistochemistry (Figure 7D). These findings show that knockdown of hMAGEA2 critically repressed the progression of TNBC.

## DISCUSSION

MAGE protein families are highly expressed in a wide range of cancers, including breast, ovary, lung, melanoma, pancreas, and bladder cancer [38–40], and have been found to act as an oncogenic driver. The expression of MAGEA3 stimulates cell cycle progression and rate of migration and invasion in thyroid cells *in vitro*; these characteristics are associated with aggressive behavior in cancer cells [38]. Silencing MAGEB in murine melanoma cells suppresses melanoma growth in xenografts [39]. Numerous studies show that the expression of MAGEA2 can promote additional characteristics associated with malignancy. MAGEA2 confers resistance to clinically relevant chemotherapeutic drugs and promotes a phenotype associated with cancer progression. Studies indicate that hMAGEA2 interacts with key proteins, notably p53 tumor suppressor protein, and affects the progression and relapse of breast cancer. hMAGEA2 was shown to complex directly with p53 and recruit HDAC3 to repress transcriptional activity of p53 [36, 40–42].

Firstly, we investigated the expression of hMAGEA2 in human TNBC tissues and compared it with that in precancer tissues. Several studies indicated that hMAGEA2 was not expressed in normal breast tissue and even normal portions of precancer tissue show no expression of hMAGEA2 [19, 43]; therefore, we did not examine the expression of hMAGEA2 in normal breast tissue. Generally, precancer tissue shows a slightly disordered cellular morphology that is associated with increased risk of cancer; this is defined as potentially premalignant condition. We found that hMAGEA2 was significantly overexpressed in human TNBC and breast cancer tissues. Furthermore, we confirmed that hMAGEA2 is overexpressed in human prostate cancer tissues (data not shown). Numerous reports show that hMAGEA2 is overexpressed in cancer tissues [38–40]; our results indicate that hMAGEA2 plays a role in TNBC and breast cancer and can be used as a clinical prognostic marker.

To confirm the function of hMAGEA2 in TNBC cell lines, we established stable overexpression of hMAGEA2 in the MDA-MB-231 and MDA-MB-468 TNBC cell lines. hMAGEA2 was strongly overexpressed in MDA-MB-231 cell line; however, in MDA-MB-468 cell line, hMAGEA2 was not significantly overexpressed at the level of protein expression. It is possible that the endogenous expression of *hMAGEA2* was already at the level of saturation, keeping the expression of hMAGEA2 protein stable in MDA-MB-468 cell line. The phenotype of the MDA-MB-468 cell line was not altered by overexpression of hMAGEA2; therefore, the knockdown of hMAGEA2 was conducted in MDA-MB-468 cell line. Cell proliferation, anchorage-independent colony formation, migration, and ability to invade are important characteristics in the phenotype of cancer cells; therefore, numerous studies assess these phenotypic traits [44–46]. We showed that overexpression of hMAGEA2 in MDA-MB-231 cell line led to a significantly increased cellular proliferation and upregulated levels of Ki-67. Anchorage-independent colony formation was also enhanced, but ability to migrate was unchanged by overexpression of hMAGEA2. Conversely, the knockdown of hMAGEA2 had the opposite effect on MDA-MB-468 cell line. To confirm metastatic ability, we performed an invasion assay in TNBC overexpressing hMAGEA2. The process of metastasis requires cancer cells to leave their primary tumor and acquire migratory and invasive capabilities. In the process of epithelial-mesenchymal transition (EMT), cancer cells change their adhesive repertoire and employ developmental processes to gain migratory and invasive properties that involve a dramatic reorganization of the actin cytoskeleton and concomitant formation of membrane protrusion required for invasive growth. Therefore, the loss of epithelial marker E-cadherin and expression of mesenchymal marker vimentin are major hallmarks of EMT [47, 48]. Our results show that the expression of E-cadherin decreased and that of vimentin increased when hMAGEA2 was overexpressed. The number of invading cells also increased with overexpression of hMAGEA2. These findings suggest that hMAGEA2 is critically associated with metastasis of TNBC via mechanisms involved in EMT. In previous studies, EMT is involved in many transcription factors. Recently, we have been studying and, according to the study, increased expression of Snail1 and TGF-β2 among many transcription factors is observed, and it is presumed that it is closely related to *hMAGEA2* gene (data not shown). However, the specific mechanism underlying the effects of hMAGEA2 on the process of EMT remains unknown and requires further investigation.

It is possible that hMAGEA2 exerts its oncogenic effects via stimulation of various signaling pathways. Previous studies indicate that MAGEA proteins inhibit transactivation of p53 by recruiting histone deacetylase 3 (HDAC3) to sites of p53 interaction with promoters, leading to resistance to anticancer drugs such as etoposide [34–36]. Therefore, we initially predicted that p53 was related to the progression of TNBC. However, contrary to our hypothesis, MDA-MB-231 and MDA-MB-468 cell line endogenously express a mutation in p53 [49]. A mutation in p53 does not function as a tumor suppressor [50, 51]. Additionally, we identified MDM2 protein expression that regulates p53 protein. However, there was no significant difference in protein expression (data not shown). Therefore, we excluded the role of p53 and examined other signaling pathways. Akt and Erk1/2 are important signaling proteins in the progression of TNBC and other cancers [7]. Previous studies show that TNBC is characterized by increased phosphorylation of Akt [14]. Moreover, ERK is a valuable prognostic marker and may be an important therapeutic target in the treatment of TNBC [15]. Our data shows that phospho-Akt and phospho-Erk1/2 were significantly activated by overexpression of hMAGEA2. Conversely, via knockdown of hMAGEA2 in MDA-MB-468 cell line, we also confirmed that hMAGEA2 significantly regulated the expression of phospho-Akt and phospho-Erk1/2. Additionally, knockdown of hMAGEA2 downregulated the expression of MAP kinase phospho-p38; the oncogenic potential of p38 has been shown in several studies [52–54]. These findings indicate that hMAGEA2 may regulate Akt and Erk1/2 upstream of the Akt and Erk1/2 signaling pathway. Additionally, There is a porssibility that the mechanism of hMAGEA2 affects MDA-MB-231 cell line and MDA-MB-468 cell lines. Further studies are needed to uncover the mechanism by which hMAGEA2 regulates the activation of Akt and Erk1/2.

Our analysis of tumorigenesis, performed *in vivo*, indicated that overexpression of hMAGEA2 accelerated, whereas the knockdown of hMAGEA2 inhibited, tumor growth. These findings suggest that hMAGEA2 sufficiently affects tumor formation and growth *in vivo*. Additionally, we examined phosphorylation of Akt and Erk1/2 in tumor tissue. Analysis using immunofluorescence confirmed that hMAGEA2 strongly induced the expression of phospho-Akt and phospho-Erk1/2 in xenograft tumor tissues.

Understanding of the molecular mechanisms involved in TNBC will enable the development of more effective treatment strategies. We identified that hMAGEA2 plays a critical role in the progression of TNBC. We also demonstrated that the expression of hMAGEA2 in human TNBC tissues was associated with accelerated proliferation, anchorage-independent colony formation, and EMT-mediated metastasis. Furthermore, hMAGEA2 led to a significant increase in the phosphorylation of Akt and Erk1/2; the activity of Akt and Erk1/2 is directly implicated in the progression of TNBC. In a recent study, Hurst and colleagues indicate that the overall survival rate of breast cancer patients is much lower in those with hMAGEA2-positive tumor tissue [33]. And although not yet reported, an inhibitor that can effectively inhibit hMAGEA2 protein may be a new target of anticancer drug. In summary, hMAGEA2 can be used as a prognostic marker in the treatment of TNBC. Our study may provide a new therapeutic target and enhance the understanding of molecular mechanisms underlying TNBC and other cancers.

## MATERIALS AND METHODS

### Construction of tissue microarray and immunohistochemistry

The tissue microarrays (TMAs) were constructed with 1 mm diameter core punched two to three distinct regions from each formalin-fixed paraffin-embedded (FFPE) tumor block. The TMAs were assembled with a tissue arrayer (Beecher Instruments, Silver Spring, MD). One section from each TMA was stained with hematoxylin and eosin (H&E) stain and reviewed to confirm the presence of representative tumors. After deparaffinization, rehydration, and antigen retrieval, the localization of hMAGEA2 in TMAs was assessed by immunohistochemistry (IHC) using antibodies (sc-130164, Santa Cruz) and the Ultraview Universal DAB Detection Kit (Ventana, Tucson, AZ). All sections were counterstained with hematoxylin. TMA sections were assessed for the intensity of the stain and the actual percentage of stained cells in the nucleus, cytoplasm, and cell membrane. Staining was considered positive when there was moderate or strong immune reactivity at the appropriate location over the cut-off point. A sample of 25 patients with breast cancer was used and 5 patients with TNBC prognosis.

### Cell culture and transfection

The human TNBC cell lines, MDA-MB-231 and MDA-MB-468, were cultured in Dulbecco's modified Eagle's medium (DMEM, Gibco, Life Technologies, Grand Island, NY, USA) supplemented with 10 % fetal bovine serum (FBS, Gibco, Life Technologies, Grand Island, NY, USA) and 1 % penicillin/streptomycin (P/S, Gibco, Life Technologies, Grand Island, NY, USA). For stable expression of hMAGEA2 in TNBC cell lines, cells were seeded into 100-mm dishes. After 24 h, cells were transfected with pcDNA3.1-mock or pcDNA3.1-hMAGEA2 vector using FuGENE HD (Promega, Madison, WI) transfection reagent following the manufacturer's instructions. To establish stable expression, the transfected TNBC cell lines were treated with G418 (1.5 mg/ml) for 7 days. Then, subcultures were treated with G418 every 3 days. All cell lines were maintained at 37°C in a 5 % CO_2_ incubator.

### Cell proliferation (CCK-8) assay

Cell proliferation was estimated using the CCK-8 assay (Cell Counting Kit-8, Dojindo Molecular Technologies, Inc.). Cells were seeded into 96-well plates (1 × 10^3^ cells per well) and incubated for 0, 24, 48, and 72 h. In total, 10 μl of CCK-8 solution was added to each well and incubated for an additional 1 h at 37°C in a 5 % CO_2_ incubator. The optical density (OD) of each well was measured at 450 nm using a spectrophotometer (BioTek).

### Anchorage-independent colony formation assay

The effects of hMAGEA2 on anchorage-independent growth were investigated in the TNBC cell lines. Cells (8 × 10^3^ cells per ml), suspended in DMEM supplemented with 10 % FBS and 1 % P/S, were added to the top layer of 0.3% agar (Top agar), over a base layer of 0.5% agar (Bottom agar). The cells were cultured at 37°C in a 5% CO_2_ incubator for 2–3 weeks. The number of colonies was counted using a microscope (Leica), and the diameter of each colony was measured.

### Matrigel invasion assay

Matrigel invasion assays were performed in 24-well transwell plates (Corning) using Matrigel (BD Biosciences) according to the manufacturer's instructions. Cells (5 × 10^4^ cell per well) were seeded into the upper chamber containing serum-free DMEM. The lower chamber was filled with complete DMEM medium. After 24 h, cells that had invaded the Matrigel were stained with crystal violet (Sigma). All experiments were conducted in triplicate.

### Lentiviral production and infection

The lentiviral hMAGEA2 shRNA vectors (shMAGEA2 sequence: 5′-GATAATCGTCCTGGCC ATAAT-3′) for the knockdown of hMAGEA2 were purchased from Sigma. Oligonucleotides were cloned into the pLKO.1 lentiviral vector. HEK293T cells were co-transfected with pLKO.1-scramble or pLKO.1-shMAGEA2 and pMDLg/p RRE, pMD2.G, and pRSV-Rev using FuGENE HD transfection reagent (Promega). The MDA-MB-468 cells were infected with lentiviruses encoding shRNA using 8 μg/mL protamine sulfate (Sigma). After 48 h, cells were selected by puromycin (1 μg/ml) for 4 days, establishing a stable knockdown of hMAGEA2 in the MDA-MB-468 cell line.

### Xenograft tumor formation assay

All procedures involving animals were performed in accordance with guidelines and approval of the Kyungbook National University. Balb/c female nude mice were injected subcutaneously in the flank with 200 μl of cells (1 × 10^7^ cells) suspended in phosphate buffered saline (PBS). Approximately 3 weeks after injection, tumor tissues were extracted from the nude mice for histological analysis, immunofluorescence, and Western blot.

### Immunohistochemistry

The tumor tissues extracted from nude mice were fixed in 4% paraformaldehyde at 4°C overnight, embedded in paraffin, and sectioned at the thickness of 7 μm. Sections were stained with H&E, after which immunohistochemistry was performed using human MAGE-A2 as the primary antibody (sc-130164, Santa Cruz).

### Immunofluorescence

Paraffin sections were deparaffinized, washed in PBS, and subjected to antigen retrieval. Sections were incubated with the primary antibody at 4°C overnight, then with the secondary antibody for 2 h at room temperature (RT), then mounted with VECTASHIELD Antifade Mounting Medium with DAPI (H-1200-10; Vector Laboratories, Burlingame, CA, USA). We used the following primary antibodies: anti-phospho-Akt (Ser473)(#9271, Cell Signaling Technology) and anti-phospho-Erk1/2 (Thr202/Tyr204)(#9101, Cell Signaling Technology). Representative images were captured using a fluorescence microscope (Leica).

### Real-time PCR

Total RNA was harvested from cells using TRIzol reagent (Invitrogen) according to the manufacturer's instructions. cDNA was synthesized using an RT-PCR kit (TAKARA, Tokyo, Japan) and measured using qPCR with SYBR Premix EX Taq (TAKARA). To assess the expression of human *MAGEA2*, proliferation marker *Ki-67*, and the anti-apoptosis marker survivin, real-time PCR was performed using Step One Plus PCR system (Applied Biosystems, Foster City, CA, USA). Relative expression was quantified and normalized using relative expression of -actin per each sample, respectively.

### Western blot

Lysis buffer (Intron Biotechnology, Seongnam, Korea) was used for protein extraction in cells or tumor tissues. The primary antibodies were human MAGEA2 (sc-130164, Santa Cruz), phospho-Akt (Ser473)(#9271, Cell Signaling Technology), Akt (#9272, Cell Signaling Technology), phospho-Erk1/2 (Thr202/Tyr204)(#9101, Cell Signaling Technology), Erk1/2 (#4695, Cell Signaling Technology), phospho-JNK (Thr183/Tyr185)(#9251, Cell Signaling Technology), JNK (#9252, Cell Signaling Technology), phospho-p38 (Thr180/Tyr182)(#4511, Cell Signaling Technology), p38 (#8690, Cell Signaling Technology), and β-actin (sc-47778, Santa Cruz). Nitrocellulose membranes were incubated overnight at 4°C with primary antibodies diluted in 5% skim milk or bovine serum albumin (BSA). Secondary antibodies, anti-rabbit and anti-mouse IgG, labeled with horseradish peroxidase (HRP) (Thermo Scientific, Seoul, Korea), were diluted in 5% skim milk or BSA; nitrocellulose membranes were incubated with the secondary antibodies for 2 h at room temperature. Development was performed using electrochemiluminescence substrate (GE Healthcare, Seoul, Korea) and protein expression was visualized using the DaVinci software.

### Statistical analysis

All data are presented as means ± SD of triplicate samples from at least three independent experiments. Differences between means were assessed using ANOVA; *p* ≤ 0.05 was considered statistically significant.
